# Substrate Specificity Checkpoints of the Multidrug Efflux Pump MexF from *Pseudomonas aeruginosa*

**DOI:** 10.1021/acsinfecdis.5c00760

**Published:** 2026-01-16

**Authors:** Muhammad R. Uddin, Silvia Gervasoni, Giuliano Malloci, Paolo Ruggerone, Helen I. Zgurskaya

**Affiliations:** † Department of Chemistry and Biochemistry, 6187 University of Oklahoma, 101 Stephenson Parkway, Norman, Oklahoma 73019, United States; ‡ Department of Physics, 3111 University of Cagliari, Monserrato, 09042 Cagliari, Italy

**Keywords:** *Pseudomonas aeruginosa*, multidrug efflux, transporter mechanism, substrate specificity, molecular docking

## Abstract

Multidrug efflux pumps of the resistance-nodulation-division (RND) superfamily are major contributors to antibiotic resistance in *Pseudomonas aeruginosa*. Among these, the MexEF–OprN system, when overproduced in clinical isolates, confers resistance to fluoroquinolones, trimethoprim, and chloramphenicol. The inner-membrane RND transporter MexF in this complex exhibits a relatively narrow substrate specificity and the molecular mechanisms underlying this specificity are still unclear. Here, we employed a combination of experimental and computational approaches to dissect the role of a major putative recognition/binding site, the Access pocket, in the substrate specificity of MexF. Mutations at four selected positions D132, P136, G626, and S729 altered resistance profiles and substrate specificity in a residue- and substrate-specific manner. Notably, substitutions at P136 enhanced efflux of most tested antibiotics, among which are 21 fluoroquinolones with different structures. Substitutions in S729, on the other hand, either enhanced or severely impaired MexF activity depending on the substitution. Antibiotic substrates were found to compete with a fluorescent probe for MexF efflux revealing overlapping binding determinants and shared translocation paths within the transporter. Ensemble docking and contact frequency analyses further demonstrated that mutations reshaped ligand binding preferences within the periplasmic cleft, modulating the probability of transition to the Deep pocket and subsequent extrusion. Our results demonstrate that MexF is optimized to trimethoprim-like compounds and single substitutions in key residues can dramatically change the substrate spectrum of this pump. These findings underline the importance of not only static binding contacts between substrates and a polyspecific transporter such as MexF but also spatial occupancy and pathway integrity in determining drug efflux efficiency.

## Graphical abstract

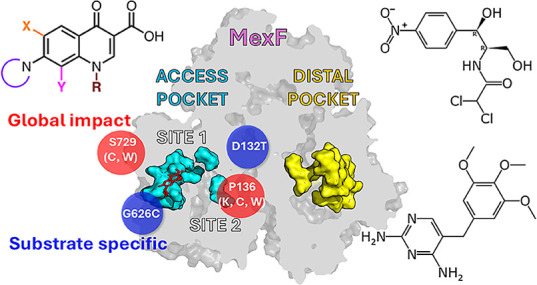

*Pseudomonas aeruginosa* is a notorious opportunistic pathogen responsible for a wide range of infections in humans, particularly in immunocompromised individuals and those with compromised epithelial barriers.^1^ Its ability to evade antibiotics is due to a complex array of resistance mechanisms, including the overexpression of multidrug efflux pumps, which actively remove chemically different toxic compounds from the bacterial cell.^2^ Among these, the Resistance-Nodulation-Division (RND) superfamily of efflux transporters plays a significant role in multidrug resistance (MDR) by expelling a broad range of antibiotics.^3,4^ MexAB–OprM and MexEF–OprN are the RND efflux systems most frequently overproduced in *P. aeruginosa* clinical isolates, with the MexEF–OprN pump playing a pivotal role in MDR against fluoroquinolones (FQs), trimethoprim (TMP), and chloramphenicol (CHL), particularly in cystic fibrosis (CF) patients.^5^ On the other hand, recent studies of clinical isolates showed that MexEF–OprN is frequently mutated with 53.96% of Intensive Care Unit (ICU) isolates and 40.8% of CF isolates having nonsynonymous mutations in mexE, mexF, or oprN.^6^ In addition, some isolates contain inactivating mutations in this pump, which are also associated with increased *P. aeruginosa* virulence and higher mortality rates among patients due to the elevated quorum sensing in the *mexEF–oprN* mutants.

MexEF–OprN is a tripartite complex, consisting of the inner-membrane transporter MexF, the periplasmic membrane fusion protein (MFP) MexE, and the outer-membrane factor OprN.^3,7^ The periplasmic MexE links MexF transporter to the OprN channel located in the outer membrane.^8^ Together, these proteins form a continuous complex across the bacterial inner and outer membranes that enables efficient efflux of substrates directly into the external environment.^9–11^ MexF plays a pivotal role in the function of the MexEF–OprN by recognizing antibiotics and by transmitting conformational changes to MexE and OprN during substrate transport.^7,9^

Unlike MexAB–OprM, the major efflux pump of *P. aeruginosa* which is constitutively expressed under laboratory conditions and during infections, MexEF–OprN is typically dormant but is frequently upregulated by mutations in regulatory pathways in response to antibiotic stress.^12^ Recent studies showed that while MexF exhibits a narrower substrate specificity than MexB, it provides higher resistance levels to FQs, TMP, and CHL.^13^ The minimum inhibitory concentrations (MICs) for these antibiotics increase dramatically when the MexEF–OprN system is overexpressed, surpassing the resistance levels conferred by MexB.^13^

A large body of available structural information about RND transporters and their tripartite complexes does not reveal the differences that could account for diverse substrate specificities and efflux efficiencies of these transporters. Most RND type transporters exhibit an asymmetric trimeric structure, where each protomer features three distinct domains: (1) the 12 α-helices transmembrane domain harnessing the energy from proton-motive force to drive substrate transport; (2) the pore domain in the periplasm, responsible for substrate recognition, binding and transport; and (3) the periplasmic funnel domain connected to the outer-membrane channel via MFPs.^3,14,15^ According to experimental and computational studies,^7,15,16^ the extrusion of compounds by RND transporters follows the so-called “functional rotation mechanism”, where protomers alternate cyclically through three asymmetric states: loose (L or Access), where substrates bind to the Access pocket (AP); tight (T or Binding), where substrates interact with the Deep pocket (DP); and open (O or Extrusion), where substrates are expelled through the periplasmic funnel and into the extracellular medium via associated outer-membrane channels. The AP and DP binding pockets are thought to play crucial roles in substrates recognition and selection by RND pumps.^7,17^ These two pockets are separated by a flexible G-rich switch loop, which facilitates the transport of larger molecules from the AP to the DP.^18^ Additionally, a group of phenylalanine residues within the DP, referred to as “the hydrophobic trap” (HP-trap), interact with substrates and stabilize inhibitors in the transporter’s periplasmic region.^19^

The MexF crystal structure remains unresolved, but the structures of two of its close homologues OqxB and BpeF from the same phylogenetic branch of RNDs have recently been published.^20,21^ These studies highlighted several structural differences in the binding sites and in the G-rich loop between OqxB/BpeF/MexF and the constitutively expressed MexB and its close homologues. In addition, computational approaches have previously been employed to construct all-atom models of MexF and to compare various *P. aeruginosa* Mex efflux pumps.^3^ These studies suggested that despite the structural particularities of the Mex transporters, they share the overall “topology” of the binding sites, which accounts for redundancy but allows subtle sequence alterations at specific sites to confer different binding abilities to each of them.

In this study, using a combination of experimental and computational approaches, we investigated the substrate specificity of MexF against a series of structurally similar antibiotics from the fluoroquinolone class as well as other representative antibiotics that vary in their sizes and mechanisms of actions. We found that mutations of a few key residues altered ligand binding preferences within the periplasmic cleft, influencing the likelihood of a transition to the DP and subsequent extrusion. Our study highlights that, beyond static binding interactions with a polyspecific transporter like MexF, factors such as spatial occupancy and the continuity of the binding pathway play a crucial role in determining efficiency of drug efflux. The interplay between all of these factors, including long-range effects, remains a great challenge in designing compounds able to counteract the efflux mechanism.

## Results

### Mutations Altering Substrate Specificity of MexF

To elucidate the molecular determinants governing substrate specificity in MexF, we conducted targeted site-directed mutagenesis of four nonconserved residues located along the proposed substrate translocation pathway of RND transporters.^22^ The selected residues—Ser729, Asp132, Pro136, and Gly626—occupy strategic positions within the transporter’s periplasmic cleft, corresponding to the AP (S729), the interface between AP and DP (D132 and P136), and the G-loop (G626) (Figure 1). These positions were chosen based on their sequence divergence and conservation from the homologous residues in the closely related transporter MexB (Asn718, Thr130, Lys134, and Phe617, respectively) (Figure S1), for which structure–function relationships have been previously characterized.^22^ Notably, interactions at these sites in MexB were shown to be predictive of both substrate recognition and inhibitor engagement.^22,23^ We engineered a series of point mutations at these positions of MexF: P136 was individually substituted with lysine (as in MexB), cysteine, or tryptophan to examine the impact of charge, polarity, and steric bulk; D132 was replaced with threonine to mimic the MexB sequence; S729 was substituted with either cysteine or tryptophan to alter the polarity and side chain volume within the inner AP; and G626 was mutated to cysteine or phenylalanine to probe the role of conformational flexibility at the base of the G-loop. Each of the resulting MexF variants was cloned into a plasmid coexpressing the periplasmic adaptor MexE and the outer-membrane channel OprN and transformed into *P. aeruginosa* Δ4-Pore (Δ*mexAB-OprM* Δ*mexXY* Δ*mexCD-OprJ* Δ*mexJKL*), a strain lacking the four major RND efflux systems.^24^ Western blot analysis of membrane fractions confirmed that all mutant MexF variants, except for G626F that was not expressed, were stably expressed and localized correctly to the membrane (Figure S2).

**Figure 1:**
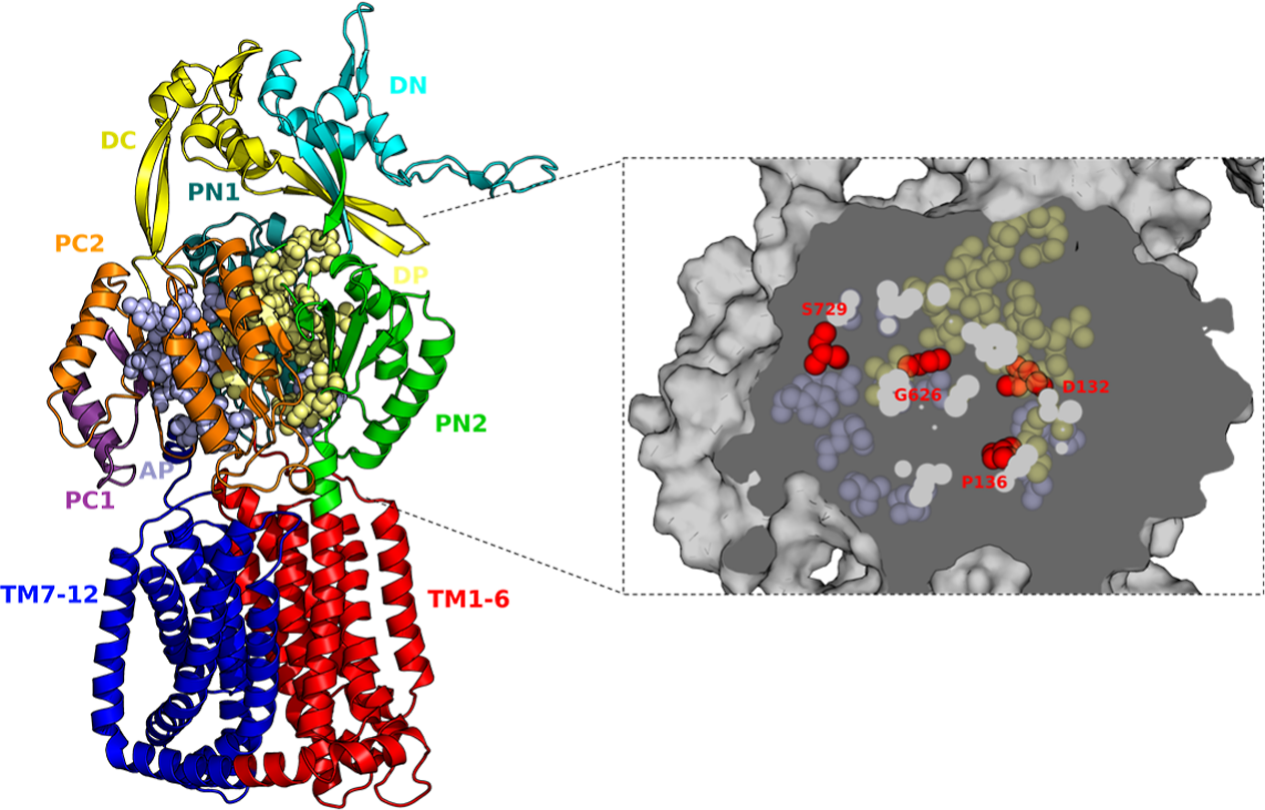
Mapping functional hotspots in MexF reveals distributed substrate binding interfaces. Homology model of MexF. Mutational sites are shown as red spheres, with Access pocket (AP, pale violet spheres) and Deep pocket residues (DP, pale yellow spheres) emphasizing the multidomain nature of substrate interaction. Transmembrane (TM1-6/TM7-12), periplasmic (PC1/PC1, PN1/PN2), and docking (DC/DN) domains are highlighted.

To evaluate how specific substitutions in MexF affect its functional activity, we next determined the MICs of representative antibiotics from different classes in *P. aeruginosa* Δ4-Pore cells overexpressing MexEF–OprN variants (Table 1, the structures of antibiotics are shown in Table S1). All tested mutants, except for G626F, retained efflux functionality; however, they exhibited distinct and residue-specific changes in the substrate selectivity. All three substitutions at Pro136 (P136K, P136C, and P136W), positioned at the interface between the AP and DP (Figure 1), significantly enhanced resistance to chloramphenicol (CHL), levofloxacin (LFX), doxycycline (DOX), and erythromycin (ERM) independently of physicochemical properties of the mutated residues, suggesting that this residue contributes to the gating or alignment of substrates entering the translocation pathway in a subtle way, probably through a network of interactions with close residues. Notably, these substitutions had no appreciable effect on the efflux of novobiocin (NOV) and trimethoprim (TMP), indicating that the influence of P136 is substrate-dependent. The substitution S729C, located within the inner AP, displayed a resistance profile nearly identical with the P136 mutants. In contrast, the bulky tryptophan substitution at this same position (S729W) resulted in severe attenuation of efflux activity for five of the six tested antibiotics, with LFX being the exception.

**Table 1:** Minimum Inhibitory Concentrations (MICs) of Selected Antibiotics against *P. aeruginosa*Δ4-Pore-Expressing Wild-Type (WT) or Mutant MexF Variants^a^ ^,^ ^b^

antibiotics	vector	MexF WT	P136K	P136C	P136W	G626C	S729C	S729W	D132T	no. of experiments
LFX	0.0025	0.16	5	5	5	0.625	5	0.08	0.08	*n* = 12
TMP	0.5	512	512	512	512	256	512	32	128	*n* = 8
CHL	0.5	32	128	128	128	128	128	8	16	*n* = 8
DOX	0.125	1	16	16	8	1	8	0.125	0.125	*n* = 8
NOV	4	32	64	64	32	8	64	2	4	*n* = 8
ERM	0.06	0.5	4	4	4	2	8	0.06	0.25	*n* = 8

a LFX, levofloxacin; TMP, trimethoprim; CHL, chloramphenicol; DOX, doxycycline; NOV, novobiocin; and ERM, erythromycin. The data reported are representative results from at least three independent experiments.

b MIC values (μg/mL, except LFX shown in μM) were determined using the broth microdilution method for *P. aeruginosa* Δ4-Pore cells harboring plasmids encoding WT MexF or indicated mutants, along with MexE and OprN. Values are modes based on the indicated number of experiments.

The D132T variant, mimicking the MexB residue at the AP/DP interface site, exhibited moderate reductions in efflux of CHL, DOX, and NOV. Interestingly, the G626C substitution, positioned at the base of the DP cavity, had a dual but substrate-specific effect: it enhanced efflux of CHL, LFX, and ERM but reduced the activity of MexF against TMP and NOV. Together, these results demonstrate that MexF-mediated resistance can be finely tuned by specific mutations that reshape the substrate-binding and translocation paths.

### MexF Substitutions Are Not Sensitive to Specific Chemical Scaffolds and Accommodate a Broad Range of Physicochemical Properties

Previously, we found that the specificity of MexF to fluoroquinolones (FQs) varies depending on the chemical features of these antibiotics.^24^ We next analyzed how substitutions in MexF affect MICs of a chemically diverse panel of 21 FQ antibiotics with different substituents (Table S1). In efflux-deficient control cells carrying an empty vector, all FQs demonstrated potent antibacterial activity, with MICs in the low nanomolar to subnanomolar range (Table S2). Delafloxacin emerged as the most potent compound, exhibiting subnanomolar activity. However, when WT MexF was expressed in *P. aeruginosa* Δ4-Pore cells, MICs for nearly all compounds increased by several orders of magnitude confirming the status of this class of antibiotics as MexF substrates (Table S2). One notable exception was clinafloxacin, which maintained similar levels of activity in efflux null and MexF-expressing cells, suggesting that it can avoid efflux by MexF. Clinafloxacin has a polar C-7 aminopyrrolidinyl moiety and a bulky C-8 chlorine substitution, which may interfere with the binding to MexF.

To compare different analogs, the MICs measured in cells producing MexF variants were normalized to the MICs against efflux-deficient cells carrying an empty vector (*F* = MIC_eff_/MIC_vector_) and the fold differences between the WT and mutant MexF variants (FC = *F*_mut_/*F*_wt_) were calculated for analyzed FQs and other antibiotics (Figure 2). The difference >2 fold was considered significant and interpreted as the mutant was more efficient in efflux of an antibiotic, whereas the difference <0.5 indicated that the WT was more efficient. We found that the FQs recognition by MexF is affected by both the structure of a given compound and the specific substitutions in MexF. Fleroxacin which is a trifluorinated 4-oxo-1,4-dihydroquinoline stood out because this is the only FQ, the efflux of which is not affected by substitutions in MexF, except S729W, which reduces its efflux (Figure 2). Also, weakly sensitive to substitutions in MexF is prulifloxacin (Figure 2), which is a lipophilic prodrug of ulifloxacin with a sulfur-containing four-membered ring added at C-1 and C-2 of the quinolone nucleus (Table S1). In this respect, fleroxacin and prulifloxacin are similar to TMP, which is also affected only by S729W, suggesting that these three compounds might follow specific translocation paths, which are different from those of other compounds. In addition, NOV is clearly distinct from other tested antibiotics, because its efflux is not improved by any of the substitutions, and it is reduced by G626C, S729W, and D132T (Figure 2).

**Figure 2:**
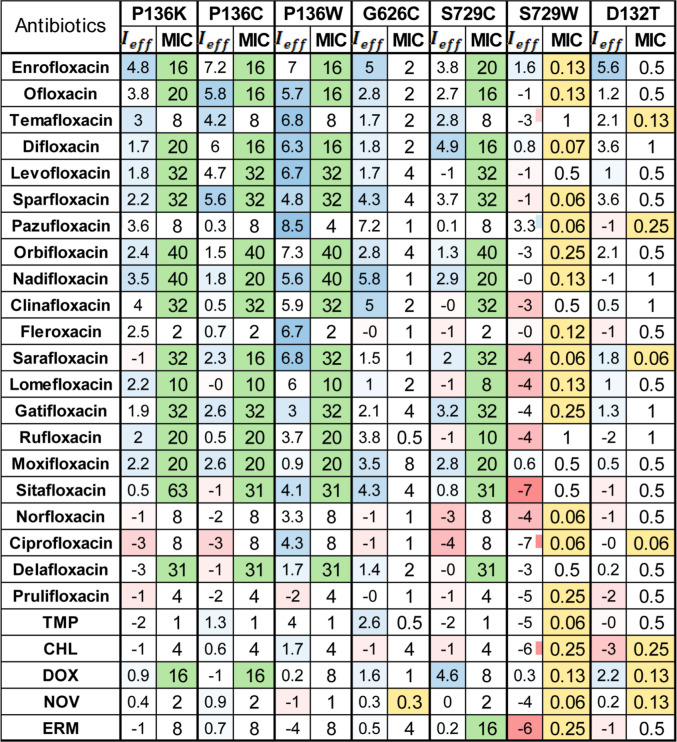
Mutation-dependent relative fold increase in MIC values of antibiotics and variation in ligand–residue contact frequencies across MexF variants. MIC values were measured in *P. aeruginosa* Δ4-Pore cells expressing wild-type or mutant MexF variants. Increased and decreased efflux with respect to the WT pump is indicated in green and yellow, respectively. The normalized contact frequency differences (*I*_eff_) between wild-type MexF and each point mutant are shown for 26 ligands. Positive values (blue) indicate higher contact frequencies in the WT, whereas negative values (red) indicate increased contacts in the mutant. Contact frequencies were derived from ensemble docking simulations targeting the AP of the loose monomer of MexF.

The recognition of all other FQs was improved by substitutions in P136 and S729C, but the effect of G626C, S729W, and D132T was specific to a given compound. Rufloxacin, which is a sulfur-linked analog of ofloxacin, and clinafloxacin (Table S1) are the only two FQs, which are not affected by G626C, S729W, or D132T substitutions (Figure 2), suggesting that their translocation paths deviate from other FQs. However, these two FQs are structurally very different and are on opposite ends of the MexF recognition spectrum, with rufloxacin being one of the best and clinafloxacin being the worst substrate of MexF (Table S2). This result further supports the hypothesis that these three MexF residues are unlikely to significantly contribute to the selection of compounds for efflux.

The recognition of only four FQs, i.e., ciprofloxacin, sarafloxacin, pazufloxacin, and temafloxacin, was strongly and negatively affected by the D132T substitution in MexF (Figure 2). In addition, the D132T variant is also defective in efflux of CHL, DOX, and NOV, which are structurally very different from FQs and each other (Table S1). This result suggests that D132 does not contribute to recognition of specific chemical features. Interestingly, temafloxacin, which is structurally similar to sarafloxacin, was able to avoid the negative effect of S729W. Perhaps, additional F- and CH_3_-modifications on the benzyl and piperazine rings make it less susceptible to the bulkier tryptophan.

Efflux of orbifloxacin, gatifloxacin, sparfloxacin, moxifloxacin, and sitafloxacin was more efficient by the MexF G626C variant. However, these FQs differed in their sensitivity to S729W. Like with temafloxacin, S729W substitution did not affect efflux of moxifloxacin and sitafloxacin, whereas efflux of orbifloxacin, gatifloxacin, and sparfloxacin was reduced by this MexF substitution. This result suggests that enhancements and reductions in MexF activities due to substitutions are not specific to compound structures. Among other tested antibiotics, efflux of CHL, the smallest (MW = 323 g/mol), and ERM, the largest (MW = 734 g/mol) among analyzed compounds, was improved by G626C substitution in MexF (Figure 2), suggesting the residue G626 at the interface between the AP and DP does not restrict the passage of the translocating substrate.

For all remaining FQs, substitutions in P136 and S729C enhanced their efflux by MexF, whereas substitution in S729W had a detrimental effect on their efflux, suggesting that all these compounds follow the same translocation path through MexF.

Therefore, our results suggest that the antibiotic efflux by MexF is shaped by distinct structural checkpoints within the main putative entry site of the transporter. With a few exceptions, mutations at P136 and S729C enhanced the export of FQs and other antibiotics, indicating that these protein residues may play a role in restricting the substrate entry and alignment within the AP. In agreement, a bulkier S729W substitution constrained the efflux of most but not all antibiotics, highlighting the flexibility of MexF. G626 and D132 contributed to substrate-specific modulation in the distal and interface regions, respectively. These findings demonstrate that the effect of substitutions is not sensitive to the specific chemical scaffolds, and even small variations in both transporter architecture and compound structure determine their efflux efficiency and resistance profiles.

### MexF Substrates Compete for the Same Binding Sites

We previously found that Hoechst 33342 (HT), a fluorescent probe which is highly fluorescent when bound to chromosomal DNA, is a substrate of MexF.^24^ We next used this assay in nongrowing cells to analyze the differences in kinetic properties of MexF variants. Here, HT was added to the cells at an increasing concentration, and the change in the fluorescence intensity was monitored in real time. As expected, due to the active efflux of HT by MexF, the steady-state accumulation levels of HT were significantly lower in cells producing MexF than in the efflux-deficient cells carrying an empty vector (Figure 3A). All mutant MexF variants were able to expel HT from the cells. However, the MexF S729W variant was notably less efficient in efflux of HT than the WT (Figure 3C) and none of the mutants demonstrated an enhanced efflux of HT.

**Figure 3:**
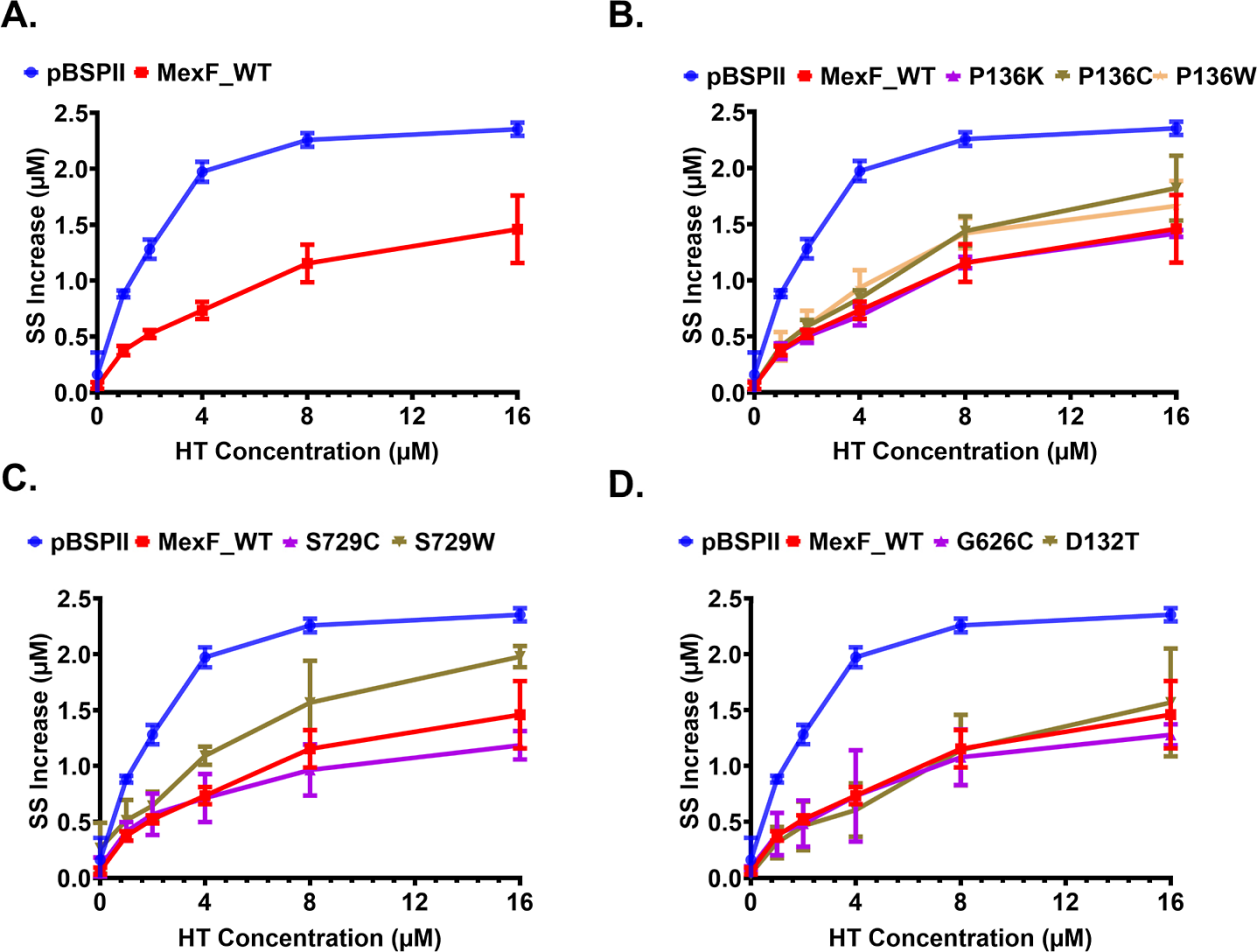
Impact of MexF mutations on efflux of the fluorescent probe Hoechst 33342. Steady-state (SS) increases in intracellular Hoechst 33342 (HT) accumulation, relative to the zero-time point, were measured in *P. aeruginosa* Δ4-Pore strains expressing plasmid-borne WT or mutant MexF variants. (A) HT accumulation in cells expressing WT MexF or empty vector (pBSPII). (B–D) HT accumulation in cells expressing the indicated MexF point mutants. Error bars are the standard deviation, SD (*n* = 3).

Since MexF has clear selectivity for some antibiotics, we next analyzed whether antibiotic substrates can compete with HT for binding to MexF in the nongrowing cells. We selected three MexF substrates, namely, TMP, CHL, and pazufloxacin (PZFX), which do not have intrinsic fluorescence overlapping with HT and were differently affected by substitutions in MexF (Figure 2). As a negative control, we included the aminoglycoside antibiotic kanamycin (KAN), which is not recognized by MexF. In these experiments, the external concentration of HT was kept constant at 4 μM and increasing concentrations of antibiotics were added to the cells. If an antibiotic can compete with HT for MexF binding sites, we expect to see an increase in the steady-state accumulation levels of HT in MexF-producing but not in efflux-deficient cells. We found that all three MexF substrates, TMP, CHL, and PZFX, but not KAN, can inhibit the MexF-dependent efflux of HT, with TMP being the most effective inhibitor (Figure 4). This result suggests that various MexF substrates share at least some interactions within the MexF binding pocket.

**Figure 4:**
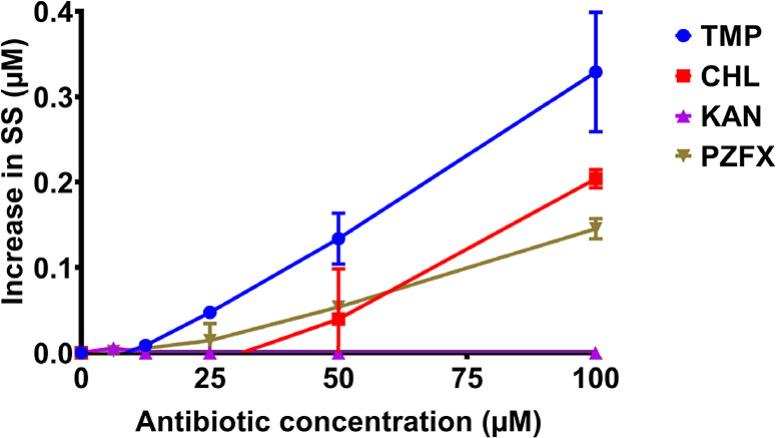
Competitive inhibition of HT efflux suggests shared binding sites in MexF. Efflux competition was assessed in nongrowing *P. aeruginosa* Δ4-Pore cells expressing MexF. Cells were treated with 4 μM Hoechst 33342 (HT) and increasing concentrations (0–100 μM) of the indicated antibiotics. The *Y*-axis shows the normalized increase in steady-state intracellular HT fluorescence relative to the zero-time point. Error bars are the standard deviation, SD (*n* = 3).

To analyze how substitutions in MexF affect the ability of TMP to inhibit the efflux of HT, experiments were carried out with cells producing different MexF variants (Figure 5). We found that the effect was due to both the amino acid position and the type of substitution. The MexF G626C mutant was very similar to the WT with half-inhibitory concentrations IC_50_ = 59.2 ± 1.6 and 65.5 ± 12.6 μM, respectively (Table S3 and Figure 5). Thus, the activity of TMP is not sensitive to this substitution, and this substitution does not affect interactions of either HT or TMP with MexF (Figure 5B). The P136K, P136C, P136W, and D132T variants were more sensitive to inhibition by TMP with IC_50_ values decreasing to 27.0 ± 8.4, 18.6 ± 3.8, 18.0 ± 4.8 μM, and 9.4 ± 7.2 μM, respectively, suggesting that these substitutions increased the affinity to TMP. These substitutions, however, did not affect the efflux of TMP in the MIC assays (Figure 2) and negatively affected the efflux of HT in nongrowing cells but only weakly (Figure 3). Thus, in these mutants, the translocation of HT enhances the interactions of TMP with MexF.

**Figure 5:**
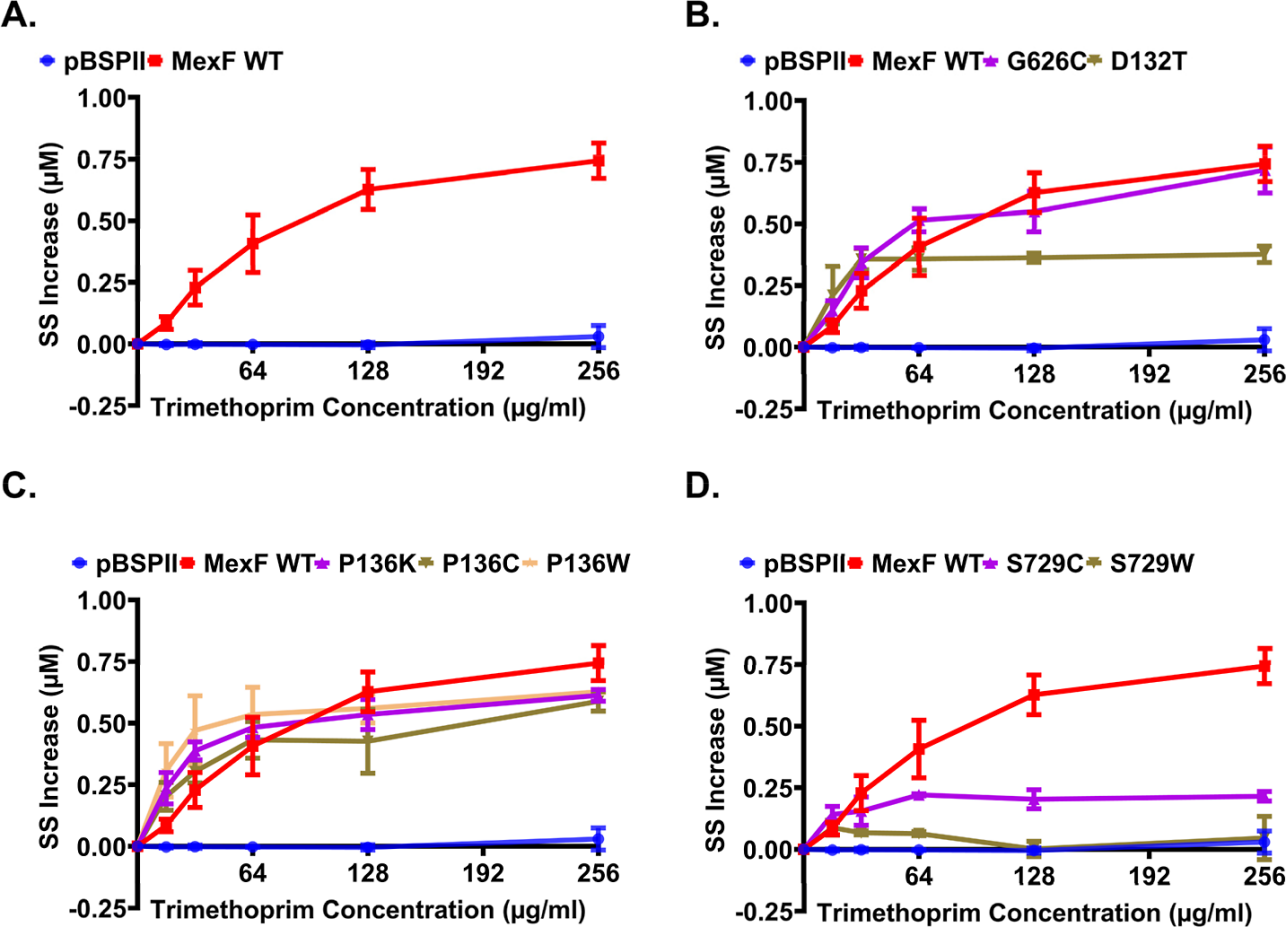
Trimethoprim competitively inhibits Hoechst efflux in a MexF variant-specific manner: (A) Steady-state (SS) accumulation of Hoechst 33342 (HT) in efflux-deficient (pBSPII) and MexF wild-type (WT)-expressing *P. aeruginosa* Δ4-Pore cells in response to increasing trimethoprim concentrations. (B–D) Dose-dependent inhibition of HT efflux by trimethoprim in MexF variants P136 K, P136C, P136W, D132T, S729C, S729W, and G626C. SS increase refers to the change in steady-state intracellular HT fluorescence relative to that in the absence of TMP (0). Error bars are the standard deviation, SD (*n* = 4).

The S729C variant showed only a small increase in the steady-state accumulation of HT suggesting that this mutant was notably less sensitive to TMP inhibition (Figure 5C). Likewise, the accumulation of HT in cells producing the S729W variant was the least sensitive to TMP inhibition and behaved as the efflux-deficient control cells with an empty vector (Figure 5D). These two substitutions, however, led to very different outcomes in MICs and HT efflux analyses. The S729C variant was more effective than WT in efflux of various antibiotics (Table 1) and as effective as WT in efflux of HT (Figure 3C). Hence, the reason for S729C insensitivity to TMP inhibition could be the better efflux of either TMP or HT. In contrast, S729W is the only MexF variant defective in protection of *P. aeruginosa* against TMP (Table 1) as well as defective in efflux of HT from nongrowing *P. aeruginosa* cells (Figure 3C).

Thus, structurally diverse MexF substrates, including TMP, CHL, and PZFX, can compete for overlapping binding sites within the transporter. The degree of competition depends on specific MexF residues and the structural and chemical features of compounds.

### Computational Analyses of Ligand Interactions Reveal Mutation-Dependent Disruption of Substrate Recognition

To gain molecular insight into ligand interactions with MexF, we performed systematic ensemble docking calculations on structural models of WT MexF and its seven D132T, G626C, S729C, S729W, P136C, P136K, and P136W variants (Figure S3).^3^ Being aware of the intrinsic approximation of molecular docking, we wanted to stress the statistical character of our computational investigation.^25^ We generated structural models of MexF (see Computational Methods), and for each target structure, we considered the AP of the loose monomer, generating a total of 600 docking poses for each run (N). This approach allowed us to capture the variability of binding events and permitted a comprehensive analysis of detailed molecular interactions at AP, for each ligand–variant pair. We performed a similar analysis for the DP of the tight monomer, but no relevant and discriminating differences for the different classes of compounds were found in this case (Table S4). We considered collectively all the generated docking poses by counting the number of contacts *C*_*j*_ between the ligand and each MexF residue *j* within a given cutoff distance (7 Å). This threshold reflects the scale of typical noncovalent interactions and enables detection of peripheral contacts that may contribute to substrate recognition/binding. The number of contacts *C*_*j*_ was then expressed in the percentage of the total number of docking poses $\left(C{\%}_{j}=\frac{C_{j}}{N}\times 100\right)$ . This number was registered for both wild-type (*C* %_wt.*j*_) and all seven MexF variants (*C* %_mut.*j*_), creating a quantitative framework for comparison of binding behavior across different transporter states.

To compare the effect of a given mutation on the interaction pattern between a selected ligand and MexF, we considered the difference Δ %_*j*_ = *C* %_*wt*.*j*_ – *C* %_mut.*j*_ associated with each residue *j*. This difference quantifies the extent to which a given mutation perturbs local contact formation with the ligand. Positive values indicate that the WT establishes more contacts with a residue as compared to the mutant, while negative values suggest that in the mutant form of the protein, either certain interactions are stronger or the ligands are trapped in alternative binding modes. For each compound, we summed all Δ %_*j*_ values associated with all contacted MexF residues, and the resulting value was normalized by the number of heavy atoms (HA) of the given compound to get an effective index:

$$I_{eff}=\frac{\Sigma \Delta {\;\%}_{j}}{HA}$$

For a given compound, the above quantity accounts for the overall change in the pattern of interactions between the WT and the mutants. This normalization allows for meaningful comparisons between ligands of different sizes and complexities, controlling for the bias introduced by larger ligands having more potential interaction points.

Figure 2 shows the values of *I*_eff_ associated with the AP for each point mutation considered. This heatmap visualization facilitates the identification of patterns where mutations either destabilize ligand binding (positive shift, blue) or promote alternative interactions (negative shift, red), supporting or challenging their functional roles observed in vivo.

To facilitate the comparison between experimental and docking results and the identification of possible trends, the computed *I*_eff_ values are shown together with the relative fold increase/decrease in MIC values (Figure 2). First, we focus on the mutations involving residues P136 and S729, which were mutated three and two times, respectively. At position 136, the three considered substitutions to lysine, cysteine, or tryptophan provide the appropriate framework to unveil the role of the mere position of the residue and/or its physicochemical properties. Compounds listed in Figure 2 can be partitioned into three main categories: (1) those with a number of contacts always larger in WT (positive *I*_eff_); (2) those with a number of contacts larger in the mutated forms (negative *I*_eff_); and (3) those in an intermediate situation. We can see that most Category 1 compounds (WT > mutant) are largely affected by the mutations, with fold-changes greater than 8, independently of the nature of the mutations. The only exceptions in this category are fleroxacin, pazufloxacin, and temafloxacin. The same trend can be observed for sarafloxacin, sitafloxacin, lomefloxacin, and DOX with mixed behavior (Category 3), for which two out of three *I*_eff_ indexes are positive, indicating a larger number of contacts in the WT. Notable exceptions in Category 3 are TMP and NOV, the activities of which are not affected by mutations in P136. Most Category 2 compounds (mutant > WT), i.e., prulifloxacin, ciprofloxacin, norfloxacin, and ERM, exhibited fold-changes less than or equal to 8, with the only relevant exception being delafloxacin, which is largely affected by the three mutations (Figure 2).

The comparison of S729C and S729W variants did not reveal any simple correlations between the number of contacts in the mutant MexF variants and the impact on the efflux of these compounds. Most tested antibiotics had a higher negative difference in contacts (negative values of *I*_eff_) between S729W and WT than between S729C and WT (Figure 2), which correlates with the experimental data that the S729W variant is defective in efflux of most compounds. The reduced contacts in S729W point to a disruption of substrate binding that likely underlies the observed functional defect. These patterns highlight S729 as a key AP determinant for ligand recognition. However, there are exceptions to this pattern. For example, fleroxacin and levofloxacin registered no difference with the WT for both S729 substitutions, but the S729C mutation was beneficial for efflux of levofloxacin but not fleroxacin and both antibiotics were negatively affected by S729W. These exceptions suggest that these compounds bind in distinct orientations or use noncanonical interaction pathways within MexF, possibly engaging distal domains of the transporter.

Different and less clear trends can be found for the other two substitutions, G626C and D132T (Figure 2). For G626C, a fold-change in the range 4–8 was detected in the MICs values for levofloxacin, sparfloxacin, orbifloxacin, gatifloxacin, moxifloxacin, sitafloxacin, and ERM, which exhibited a reduction of contacts upon mutation (positive values of *I*_eff_). An exception is represented by CHL, which is characterized by a 4 MIC fold-change and an increased number of contacts in this MexF variant. A 2-fold increase in MICs is observed also in enrofloxacin, ofloxacin, temafloxacin, difloxacin, clinafloxacin, and lomefloxacin for which the number of contacts is larger in WT (positive values of *I*_eff_). A notable decrease in the MIC is observed for NOV, for which there is no large difference in the associated *I*_eff_, indicating a similar propensity to interact with the residue at position 626 in both WT and MexF variants. The insertion of a threonine residue in position 132 caused a meaningful reduction in the MICs of pazufloxacin, ciprofloxacin, and CHL, which are indeed characterized by more frequent contacts in position 132 upon mutation (negative values of *I*_eff_). However, the MIC reductions measured for the same substitution in the case of temafloxacin, sarafloxacin, DOX, and NOV appear not to correlate with the positive values registered for *I*_eff._

Overall, we found that the contacts extracted from docking calculations targeting the AP can discriminate against the different behaviors of the compounds and are qualitatively consistent with the experimental MIC fold-changes. This is shown by the relatively good correlation coefficients in the range 0.5–1.0 obtained between the measured MIC fold-changes associated with all mutations and the effective contact index *I*_eff_ extracted from ensemble docking experiments (53% and 15% with correlation coefficient greater than 0.5 and 0.6, respectively). Even better is the correlation if only the three mutations of residue 136 and the two of residue 729 are considered: 73% results from our docking protocol have a correlation coefficient greater than 0.5, of which 62% exhibit a correlation coefficient greater than 0.6. These findings suggest that the AP acts as a substrate-selective checkpoint and that residues like S729 and P136 play a role in filtering the compounds into the translocation pathway.

### Spatial Clustering of Ligands Reveals Mutation-Specific Remodeling of MexF’s Access Pocket

To complement the contact-based analysis of MexF–ligand interactions, we conducted a detailed structural assessment of ligand distribution patterns within the AP for WT and variant MexF proteins. We focused on the mutations of residues 136 and 729 because they offered a valid comparative framework, although data are shown for all variants. All docking poses for each of the 26 ligands considered were clustered into three groups based on root-mean-square deviation, and the center of mass of each cluster representative was mapped to evaluate spatial preferences of all compounds. This analysis permitted three-dimensional visualization of substrate localization patterns and helped link structural occupancy with experimentally observed differences in MexF efflux efficiency.

Across all proteins, most docking poses were grouped in two dominant subpockets: SITE 1, located near the AP entrance and involving residues such as P136, G626, and S729, and SITE 2, positioned deeper within the AP and abutting the entrance of DP (Table 2 and Figure 6). In WT MexF, ligand clustering showed a consistent distribution, with approximately 80% of cluster centers localized in SITE 1 and the remaining 20% in SITE 2 (Table 2). This distribution likely reflects a two-step binding trajectory wherein substrates are first captured at the AP entrance and subsequently transition toward the DP for extrusion. This model supports the moderate but functional efflux activity observed experimentally for WT MexF.

**Table 2:** Quantitative Distribution of Ligand Cluster Centers of Mass across SITE 1 and SITE 2 in MexF Wild-Type and Selected Mutants^a^

	WT	D132T	G626C	P136C	P136K	P136W	S729C	S729W
SITE 1	80	83	83	80	75	83	75	66
SITE 2	20	17	14	20	25	17	24	34

a Percentage distribution of ligand cluster centers of mass across two defined subpockets—SITE 1 and SITE 2—in the AP of MexF. The table summarizes average cluster occupancy for 26 ligands in wild-type (WT) and all variants considered. In G626C and S729C, the missing fraction of poses is located in other portions of the pocket.

**Figure 6:**
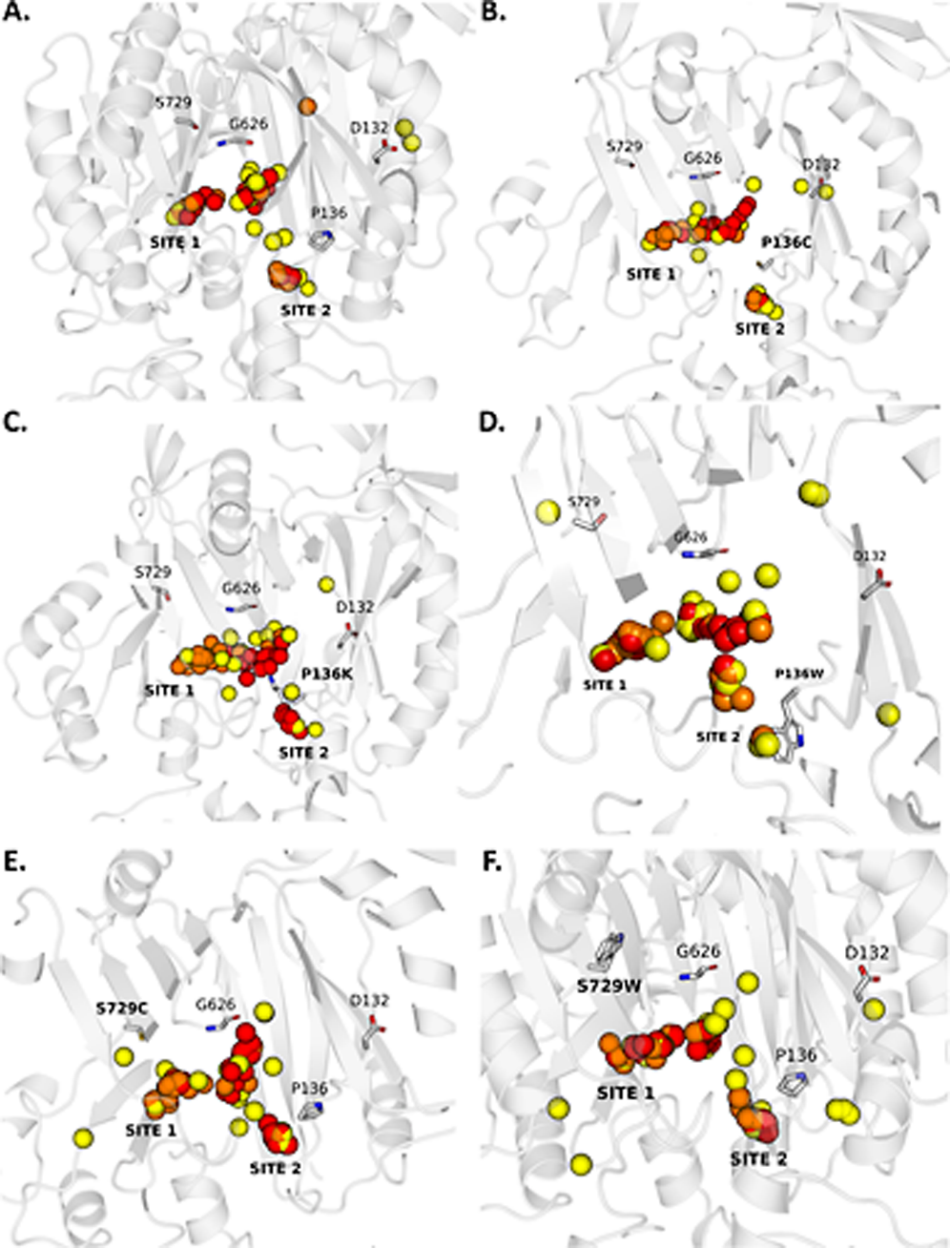
Mutation-dependent redistribution of ligand binding clusters in the Access pocket of MexF. Three-dimensional mapping of docking pose clusters for 26 ligands in the AP of MexF across (A) wild-type MexF and five key variants: (B) P136C, (C) P136 K, (D) P136W, (E) S729C, and (F) S729W. Cluster centers of mass are shown as spheres colored by cluster identity: red (cluster 0, most populated), orange (cluster 1), and yellow (cluster 2, least populated). Labels indicate two major subpockets: SITE 1 (proximal entrance) and SITE 2 (deeper near the DP interface).

In the 136 variants, ligand clustering remained identical to that of WT (80:20 SITE 1:SITE 2), indicating that substitution of proline with lysine, cysteine, or tryptophan does not significantly perturb ligand positioning, although for several compounds, a decrease in the total number of contacts around position 136 resulted from the contact maps (see Figure 2). This suggests that the local geometry and dynamics of the AP are largely preserved (Figure 6). However, the reduced frequency of contacts in the variants may improve the efflux efficacy because it can correlate with a smaller dwelling time and an improved turnover efficiency. Interestingly, an additional observation is that P136W exhibited a slightly increased preference for SITE 1 occupancy (83%), rather than the expected shift toward SITE 2 (Table 2). Despite the bulky nature of tryptophan, which could reshape the AP via hydrophobic interactions, the data indicate that ligands remain preferentially close to the AP entrance. The S729C substitution resulted in a clustering pattern of 75:24 (SITE 1:SITE 2), slightly deviating from the WT distribution. This indicates that replacement with cysteine produces minimal disruption of the AP geometry, consistent with the conservative nature of the substitution. In contrast, the S729W variant displayed a marked shift in spatial distribution, with SITE 1 occupancy reduced to 66% and SITE 2 occupancy increased to 34%. Moreover, in this case, additional cluster centers were scattered in atypical regions of the AP, indicating a disruption of the canonical ligand path toward the DP (Figure S3). This disordered distribution likely results from steric interference imposed by the bulky indole group at position 729, which may obstruct ligand alignment and impede productive engagement with the efflux axis. Functionally, this is reflected in a diminished efflux efficiency and reduced synergy in combination assays, identifying S729W as a loss-of-function mutation with respect to substrate extrusion.

Altogether, 78 cluster centers (3 per ligand across 26 ligands) were analyzed. Figure 6 visualizes these centers of mass (colored red, orange, and yellow for clusters 0, 1, and 2) mapped onto the loose monomer structure of the transporter. Comparison across WT, P136C, P136W, S729C, and S729W MexF variants reveals how specific point mutations can reorganize spatial occupancy, alter substrate trajectories, and reshape functional outcomes. These spatial distribution patterns reinforce and integrate the contact-based findings, offering a mechanistic bridge among ligand positioning, mutational effects, and efflux phenotypes. Mutations such as P136C and P136W, though not shifting ligand positioning toward SITE 2, may subtly enhance pathway efficiency by optimizing binding dynamics. Conversely, S729W disrupts the ligand orientation and spatial coherence, undermining effective substrate extrusion. According to the molecular-docking-based analysis, we postulate that the AP acts as a dynamic, mutation-sensitive conduit whose structural properties govern whether substrates proceed productively toward the DP or stall in nonfunctional poses. Further molecular-dynamics investigations are needed to deepen the details of the translocation or gating processes.

For D132T and G626C, the ligand clustering remained essentially identical to that of WT and correlation with experimental findings is not clearly recognizable. We postulate that local changes induced by these mutations might be even more subtle than those associated with the other mutations. Residue 626 is part of the G-loop, and the related mutation might affect not only its flexibility but also the transport-related interactions between the loop and the compounds in a way that is difficult to extract from docking studies. Similarly, position 132 is located at the interface AP/DP and the corresponding substitutions can alter the dynamical gating at this point, something that cannot be captured by docking alone.

## Discussion

Multidrug efflux systems are a central pillar of intrinsic and adaptive antibiotic resistance in *P. aeruginosa*, with the RND transporter MexEF–OprN playing a distinct role in virulence and the export of antibiotics under clinical conditions. Significant structural and sequence conservation between MexF and its close homologues such as *Acinetobacter baumannii* AdeG or OqxB from *Klebsiella pneumoniae* (Figure 4) on one side, and AcrB/MexB on the other, brings all these transporters into the same phylogenetic Acr cluster.^26^ However, MexF and its close homologues form a separate phylogenetic branch with divergent substrate profiles. In this study, we focused on the molecular and mechanistic basis of this divergence and identified specific residues in MexF whose modification affects substrate entry, trajectory within the transporter, and competition.

We focused on four nonconserved residues D132, P136, S729, and G626 in the AP and the interface region between AP and DP of MexF and its structural homologues. The most profound phenotypes were observed at P136 and S729, where substitutions drastically reshaped the substrate profiles. Any of the three substitutions in P136, a small Cys, a positively charged Lys, or a bulky Trp, broadened the substrate spectrum and/or improved the efflux efficiency of MexF. This residue is located at the interface between the AP and the DP and corresponds to K134 of MexB previously identified as one of the top predictors for MexB substrates and substrate specificity.^22,23^ This residue also directly interacted with several substrates in the ligand-bound structures of MexB and its homologues.^27^ Any of the three substitutions led to very similar phenotypes (Table 1 and Figure 2), suggesting that the specific side chain is not important and that removal of the constraint imposed by Pro at this position is responsible for the phenotype. Ligand binding clustering analyses showed that P136 is part of SITE 2 and that substitutions in this position do not change notably ligand distribution between the sites. Interestingly, from the contact-based analysis, we found that the largest enhancement in the efflux activity observed for the three considered mutations of P136 is mostly related to compounds that have a reduction in the contacts upon mutations, independently of the specific substitution. Combined with the results of the clustering analysis of docking poses, this suggests a central role of residue 136 in determining the subsequent steps of the transport mechanism. The absence of a dependence of the specific physicochemical properties of the mutation might indicate that the involvement of this residue is not straightforward and can involve dwelling time of the substrate within the pocket, synchronization with the other key steps of the transport mechanism (i.e., the configurational changes associated with the functional rotation), and transmission of information between the different regions of the transporters. This subtle role of residue at position 136 needs to be further explored in future studies. Interestingly, this residue is highly conserved among closed homologues of MexF but not in MexB/AcrB pumps and some of the characterized clinical isolates of *P. aeruginosa* ^6^ contain substitutions in the nearby A134 of MexF (Figure S4).

The two substitutions in S729, which is not conserved among MexF homologues (Figure S4), had dramatically different effects on the activity of MexF. The bulky side chain of S729W consistently impaired efflux of diverse compounds, while S729C conferred enhanced activity against some FQs and antibiotics. The corresponding N718 residue of MexB located in the AP directly interacts with large antibiotics such as erythromycin and doxorubicin in structural analyses, but docking analyses did not place S729 of MexF in direct contact with ligands. However, the S729W substitution shifted the spatial distribution of docked ligands, reducing the occupancy of SITE 1 and increasing it for SITE 2 (Table 2). Additional cluster centers specific for this mutant further suggest a disruption of the canonical ligand path toward the DP. The whole corpus of computational results points out that exploiting the statistics of the docking results provides a valuable tool for this kind of investigation, although we are aware of the approximation behind docking procedures. The possibility to compare the effects of different mutations associated with the same spatial location by using docking results in terms of statistical analysis allows for the mapping of those effects on the mere position in the protein, a combination of location and intrinsic features of the location, the interplay between the previous factors, and the specific chemical properties of the given compounds.

Theimpact of G626C substitution located in the G-loop of MexF was dichotomic with efflux of some substrates such as CHL, certain FQs and ERM improving but decreasing for NOV and TMP. The G-loop separating the AP and DP has been recognized previously as a contributor to substrate specificities and the role of the amino acids residues forming the G-loop has been characterized in the model RND transporters AcrB and MexB.^18,19,28^ The composition of the G-loop in MexF and its close homologues (Figure S4) is distinct from that of AcrB/MexB. The G-loop of AcrB comprises four glycine residues (G614, G616, G619, and G621), MexB three glycines (G614, G616, and G621), and only two glycines, G621 and G626 are present in the MexF loop. Interestingly, two functionally important residues F615 and F617 in the G-loop of both AcrB and MexB are responsible for blocking the substrate transport pathway.^28^ These residues are replaced in MexF with the smaller hydrophobic residues L622 and I624. It appears that not only G-loop flexibility but also the loop’s nonglycine residues are important for substrate specificities of the RND transporters. *P. aeruginosa* clinical isolates were found to contain substitutions in T628 of MexF,^6^ further vouching for the importance of the G-loop in the activity of MexF.

Direct competition assays showed that structurally diverse substrates such as TMP or PZFX can compete for the transporter and inhibit the efflux of HT (Figure 4), and the substitutions affect this competition. TMP appears to be a preferred substrate because substitutions, except S729W, did not affect its MIC values; it was the most effective competitor, and its inhibitory potency increased in P136 mutants, D132T, and G626C variants. Together with computational analyses, these findings strongly support a shared translocation pathway for structurally distinct substrates and show how specific protein mutations modify this pathway. Across the AP, MexF established more extensive contacts with substrates than most mutants, suggesting that mutations generally reduce binding affinity or alter binding site geometry. S729W disorganized clustering patterns, disrupting substrate alignment and likely preventing progression of the substrate to the DP. This shift in pose convergence suggests that S729W alters not only physical space but also the conformational dynamics needed for ligand advancement—a concept supported by cryo-EM structures showing that periplasmic helices coordinate with substrate entry to drive RND cycling.^29^ Furthermore, recent comparative mutagenesis and cryo-EM analyses of *E. coli* AcrB and *K. pneumoniae* OqxB from the MexF branch led to similar conclusions that the transfer of a single conserved residue between these two branches of RND pumps affects the resistance phenotype not only due to changes in the physicochemical properties of the binding pocket but also due to an altered equilibrium between the conformational states of the transport cycle.^30^

Higher-resolution kinetic approaches are needed to refine the effects of mutations on substrate binding affinities and the transport efficiency of MexF. Such techniques as time-resolved and single molecule fluorescence, as well as stop-flow kinetics, could provide the needed resolution. These techniques, however, are not straightforward with tripartite transporters acting across two bacterial membranes because of multiple conformational states of trimeric RND proteins and the dynamic nature of interactions with the accessory proteins.

## Experimental Procedures

### Site-Directed Mutagenesis of MexF and Protein Expression Analysis

A plasmid-borne expression of MexEF–OprN was used in this study because the parent PAO1 strain used in construction of efflux deletions contained a 7 bp deletion and two SNVs in the *mexT* gene and does not produce MexT, a transcriptional activator of the MexEF–OprN efflux pump.^31,32^ The *mexT* gene is a known hotspot and is mutated in several laboratory PAO1 strains.^33^ Site-directed substitutions were introduced into the *mexF* gene using the Q5 Site-Directed Mutagenesis Kit (New England Biolabs, cat. no. E0554S), following the manufacturer’s protocol. The plasmid pBSPII-MexEF-OprN was used as the template.^24^ The presence of the intended mutations was confirmed by DNA sequencing of the full plasmid (Plasmidsaurus). MexF variants were produced under an IPTG-inducible LAC promoter, which is not sensitive to the presence of antibiotics.^24^ Expression of MexF variants was assessed using membrane fractions prepared from cultures grown to the mid-log phase (OD_600_ ≈0.6–0.8). Proteins were resolved by SDS-PAGE and transferred to PVDF membranes for Western blot analysis. Detection was performed using a polyclonal anti-MexF primary antibody (1:50,000 dilution) and an alkaline phosphatase (AP)-conjugated antirabbit secondary antibody.

### Minimum Inhibitory Concentration (MIC) Determination

The susceptibilities of *Pseudomonas aeruginosa* PΔ4-Pore cells carrying plasmids pBSPII, pBSPII-MexEF-OprN, and various mutant variants were evaluated using the 2-fold broth microdilution method.^34^ Overnight bacterial cultures were subcultured into fresh LB broth and incubated at 37 °C shaking until the mid-log phase (OD_600_ = 0.3–0.4). The MexEF–OprN pump and pore expression were induced by adding 1 mM IPTG for 4 h. Induced cells were added to the plates containing the antibiotic dilutions and incubated at 37 °C for 18 h. MICs were defined as the lowest antibiotic concentration that inhibited visible growth. TMP, CHL, ERM, and NOV are bacteriostatic antibiotics, whereas FQs vary in their activities, with some classified as bactericidal.

### HT Uptake Assay

The HT uptake assay with *P. aeruginosa* PΔ4-Pore cells carrying plasmids (pBSPII, pBSPII-MexEF-OprN, and various MexF mutant constructs) was carried out as reported before.^24^ The cultures were diluted 1:100 in fresh LB and incubated at 37 °C until the mid-log phase (OD600 = 0.3–0.4), with MexEF–OprN expression induced by adding 1 mM IPTG for 4 h. Cells were harvested by centrifugation and resuspended in HMG buffer (50 mM HEPES, 5 mM MgCl2, and 5% glucose, pH 7.0). A black bottom 96-well plate was prepared with varying HT concentrations (0–16 μM) in HMG buffer, and bacterial suspensions (OD600 = 1.0) were added. Fluorescence was measured using a spectrophotometer (excitation: 355 nm, emission: 450 nm) before and after cell addition. Data were analyzed using MATLAB and Excel as described before.^35^ For HT efflux inhibition assays, the HT concentration was kept constant at 4 μM, and different concentrations of compounds (0–100 μM) were tested.^36^

### Computational Methods

We performed ensemble docking experiments for all 26 antibiotics targeting MexF.^3^ First, we generated a pool of MexF structures by homology modeling, exploiting good-quality X-ray structures of homologous RND transporters with high sequence identity with MexF. Then, we checked for the best docking settings through redocking experiments. Finally, we performed systematic docking runs targeting the AP_L_ and the DP_T_, for WT MexF and 7 variants (i.e., D132T, G626C, S729C, S729W, P136C, P136K, and P136W).

To generate reliable MexF structures by homology modeling with MODELER,^37^ we initially considered two template structures: (1) OqxB from *Klebsiella pneumoniae* (PDB: 7CZ9)^20^ with a sequence identity with MexF (UniProt: P95422) of 61.05%. (2) BpeF from *Burkholderia pseudomallei* (PDB: 7WLV)^21^ with a sequence identity with MexF of 63.68%. We generated three conformations of MexF using OqxB from *Klebsiella pneumoniae*, and three using BpeF from *Burkholderia pseudomallei*, ending up with six MexF structural models in total. From each one of the six MexF structures, we generated the corresponding single-point mutant with MODELER, following the standard model optimization settings of the program.

For molecular docking, the 3D structures of compounds were extracted from AB-DB.^38^ We used the docking program GOLD^39^ adopting the Goldscore scoring function. We set as the docking volume a sphere with a radius of 25.0 Å completely enclosing the binding pockets. We adopted the “very flexible” settings and generated 100 poses for each run. Contacts were made between the ligand, and we adopted the Goldscore scoring function. Docking poses and MexF were computed using PyMOL (The PyMOL Molecular Graphics System, Version 3.0 Schrödinger, LLC). The cluster analysis was performed via CPPTRAJ^40^ using a hierarchical algorithm that grouped the docking poses into three conformational clusters, according to the antibiotic root-mean-square deviation values.

## Supplementary Material



## Notes

Chemical structures of antibiotics used in this study; MIC values of 21 fluoroquinolones against *P. aeruginosa*-expressing MexF variants; IC_50_ values of trimethoprim-mediated inhibition of Hoechst efflux in *P. aeruginosa*-expressing MexF variants; mutation-dependent variation in ligand–residue contact frequencies across MexF variants for DP; pairwise alignment of MexF and MexB; expression levels of MexF and its variants; representative docking poses of selected FQs; and multiple sequence alignment of the MexF mutated regions with the closest orthologs (PDF)
