# Supplementary material for: Substrate Specificity Checkpoints of the Multidrug Efflux Pump MexF from Pseudomonas aeruginosa
Source: ACS Infect Dis. 2026 Jan 16;12(2):611–22. doi: 10.1021/acsinfecdis.5c00760 (PMC12910583; doi:10.1021/acsinfecdis.5c00760)
Supplement: Supplementary file 1 [file id5c00760_si_001.pdf]

Supporting Information

Substrate Specificity Checkpoints of the Multidrug Efflux Pump MexF from *Pseudomonas aeruginosa*

Muhammad R. Uddin[1], Silvia Gervasoni[2], Giuliano Malloci[2], Paolo Ruggerone[2,*] and Helen I. Zgurskaya[1,*]

Content:

Table S1. 2D structures of the studied antibiotics

Table S2. Efflux-dependent fluoroquinolone (FQ) susceptibility across MexF variants.

Table S3. IC_{50} values of trimethoprim-mediated inhibition of Hoechst efflux in *P. aeruginosa* Δ4-Pore strains expressing MexF variants.

Table S4: Mutation-dependent variation in ligand–residue contact frequencies across MexF variants for DP.

Figure S1. Pairwise alignment of MexF and MexB reveals divergence at substrate recognition residues within the Access and Distal Pockets.

Figure S2. Expression analysis of MexF and its variants in P. aeruginosa Δ4-Pore cells.

Figure S3. Representative docking poses of selected FQs.

Figure S4. Multiple sequence alignment of the MexF mutated region with closest orthologs.

Table S1. Chemical structures of antibiotics used in this study.

Enrofloxacin<br>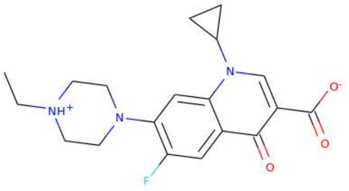	Ofloxacin<br>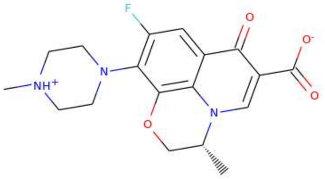	Temafloxacin<br>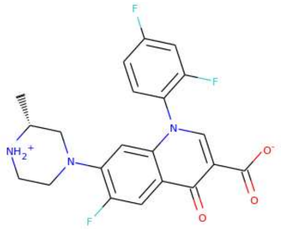
Difloxacin<br>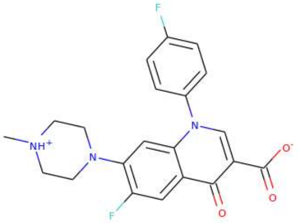	Levofloxacin<br>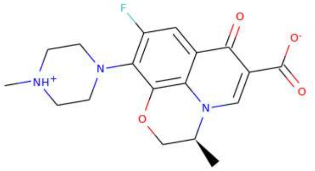	Sparfloxacin<br>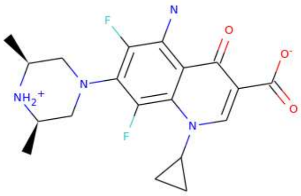
Pazufloxacin<br>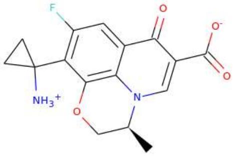	Orbifloxacin<br>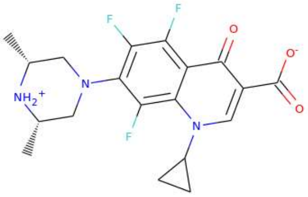	Nadifloxacin<br>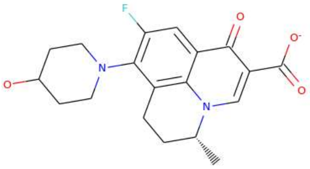
Clinafloxacin<br>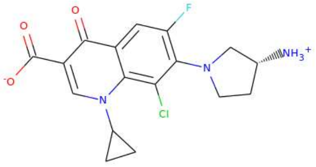	Fleroxacin<br>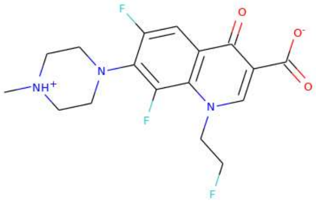	Sarafloxacin<br>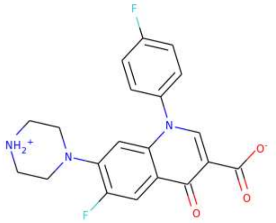
Lomefloxacin<br>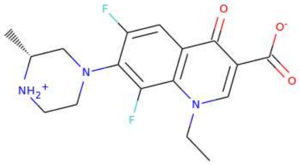	Gatifloxacin<br>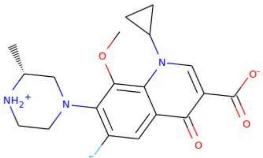	Rufloxacin<br>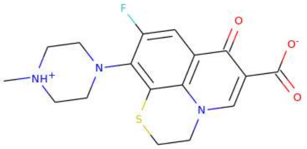
Moxifloxacin<br>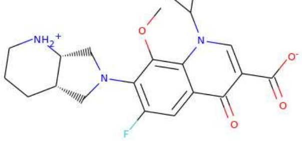	Sitafloxacin<br>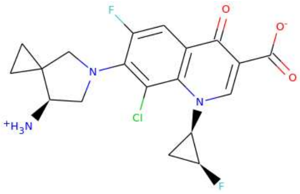	Norfloxacin<br>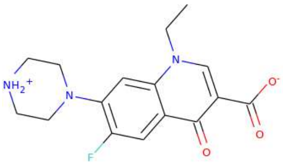
Ciprofloxacin	Delafloxacin	Prulifloxacin

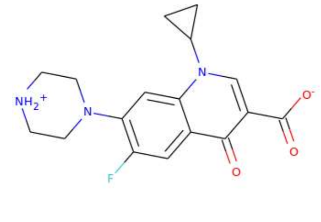	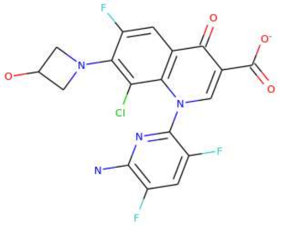	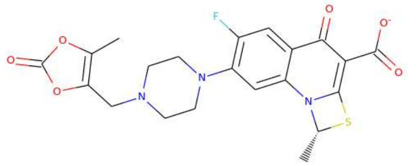
TMP 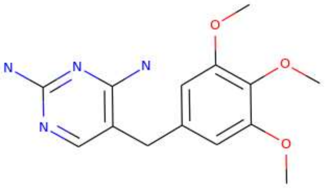	CHL 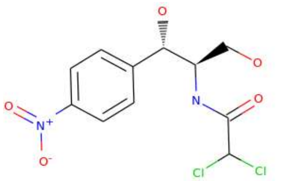	DOX 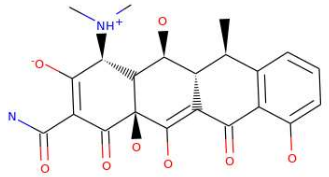
NOV 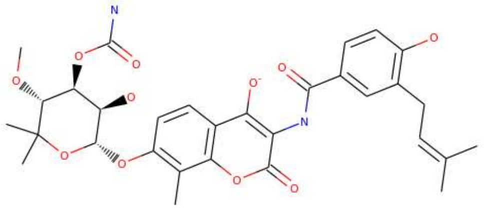		ERM 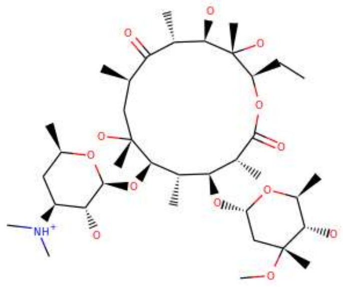

Table S2. Fluoroquinolone (FQ) susceptibility in *P. aeruginosa* producing the indicated MexF variants. MICs of 21 FQs were measured in *P. aeruginosa* Δ4-Pore cells expressing wild-type or mutant MexF, and in empty-vector expressing (pBSPII) cells. Results highlight differential efflux efficiency shaped by substrate structure and MexF mutations, particularly at P136, S729, D132, and G626. Values are modes based on the indicated number of experiments shown.

Antibiotics	pBSPII	MexF WT	P136K	P136C	P136W	G626C	S729C	S729W	D132T	No. of experiments
Rufloxacin	0.01	5	100	100	100	2.5	50	5	5	n = 4
Fleroxacin	0.01	2.5	5	5	5	2.5	5	0.313	1.25	n = 4
Orbifloxacin	0.01	0.625	25	25	25	2.5	25	0.155	0.313	n = 4
Lomefloxacin	0.005	0.625	6.25	6.25	6.25	1.25	5	0.078	0.313	n = 4
Pazufloxacin	0.003	0.625	5	5	2.5	0.625	5	0.039	0.155	n = 4
Temafloxacin	0.005	0.625	5	5	5	1.25	5	0.625	0.078	n = 4
Prulifloxacin	0.005	0.625	2.5	2.5	2.5	0.625	2.5	0.156	0.313	n = 4
Nadifloxacin	0.003	0.313	12.5	6.25	12.5	0.313	6.25	0.039	0.313	n = 4
Difloxacin hydrochloride	0.003	0.313	6.25	5	5	0.625	5	0.02	0.313	n = 4
Ofloxacin	0.005	0.313	6.25	5	5	0.625	5	0.039	0.155	n = 4
Moxifloxacin	0.003	0.313	6.25	6.25	6.25	2.5	6.25	0.155	0.155	n = 4
Enrofloxacin	0.003	0.313	5	5	5	0.625	6.25	0.039	0.155	n = 4
Norfloxacin	0.001	0.313	2.5	2.5	2.5	0.313	2.5	0.02	0.155	n = 4
Sparfloxacin	0.001	0.156	5	5	5	0.625	5	0.01	0.078	n = 4
Gatifloxacin	0.005	0.156	5	5	5	0.625	5	0.039	0.156	n = 4
Sarafloxacin	0.003	0.156	5	2.5	5	0.156	5	0.01	0.01	n = 4
Levofloxacin	0.003	0.156	5	5	5	0.625	5	0.078	0.078	n = 12
Ciprofloxacin	0.001	0.156	1.25	1.25	1.25	0.156	1.25	0.01	0.01	n = 4
Sitafloxacin	0.001	0.02	1.25	0.625	0.625	0.078	0.625	0.01	0.01	n = 4
Clinafloxacin	0.01	0.02	0.625	0.625	0.625	0.039	0.625	0.01	0.02	n = 4
Delafloxacin	<0.001	0.02	0.625	0.625	0.625	0.039	0.625	0.01	0.01	n = 4

Table S3. IC_{50} values of trimethoprim-mediated inhibition of Hoechst efflux in *P. aeruginosa* Δ4-Pore strains expressing MexF variants. Half-maximal inhibitory concentrations (IC_{50}) were determined from steady-state accumulation of Hoechst 33342 (HT) in the presence of increasing concentrations of trimethoprim. Data are shown for cells expressing wild-type MexF and the indicated point mutants. IC_{50} values reflect the concentration of trimethoprim required to reduce MexF-mediated HT efflux by 50%. "ND" indicates that an IC_{50} value could not be determined under the experimental conditions. Values are the mean (μM) of three independent biological replicates.

**Strains**	**IC_{50} (μM)**	**Standard Deviation (μM)**
pBSPII	ND	ND
MexF_WT	65.50	12.62
P136K	27.01	8.39
P136C	18.62	3.82
P136W	31.30	4.19
G626C	59.21	1.60
S729C	18.01	4.81
S729W	ND	ND
D132T	9.44	7.23

Table S4. Mutation-dependent variation in ligand–residue contact frequencies across MexF variants for DP: Heatmap summarizes the normalized contact frequency differences (Δ%j) between wild-type MexF and each point mutant (D132T, P136C, P136K, P136W, G626C, S729C, S729W) across 26 ligands. Contact frequencies were derived from ensemble docking simulations targeting the DP of the Tight monomer of MexF. As in Table 3, positive values (blue) indicate higher contact frequencies in the WT, whereas negative values (red) indicate increased contacts in the mutant.

	P136C	P136K	P136W	G626C	S729C	S729W	D132T
Fleroxacin	10.6	9.3	10.2	3.5	9.5	5.3	1.5
Prulifloxacin	7.3	6.8	6.5	3.8	4.8	4	3.2
Rufloxacin	6.8	5	7.5	-2.6	4.3	1.6	-0.6
Clinafloxacin	16.5	17.4	14.5	10.4	12.6	9.6	12.7
Pazufloxacin	13.2	11.3	17.3	11.7	13.3	10.3	9.8
Ciprofloxacin	14.4	8.7	11.4	6.5	7	7.7	4
Sarafloxacin	12.6	6	13	6.6	7.8	9.5	5.2
Temafloxacin	9.8	7.9	10.5	7.3	8.6	8.8	4.5
Moxifloxacin	8.9	7.5	8.4	2.6	9.3	7.2	5.2
Sitafloxacin	8.4	9.2	7.6	6.5	9.5	5.7	7.4
Levofloxacin	8.8	6.6	10.8	4	8	3.9	1.8
Orbifloxacin	7	5.1	9.4	3.2	8.9	6.9	5.6
Gatifloxacin	6.9	6.4	8.6	3	6.9	6	4.1
Sparfloxacin	5.1	4.8	5.1	2.6	7.3	6.6	3.8
Nadifloxacin	12.9	11.4	14.1	7	13.7	9.2	5.7
Ofloxacin	11.5	9.6	14	7.1	11.5	6.7	6.2
Norfloxacin	14.9	14.9	14.5	8.6	13.9	7.6	6.2
Lomefloxacin	13	12.3	11.2	6.9	9.4	8.2	3.3
Enrofloxacin	9.2	5.6	6.3	0.2	4.2	4.2	-1
Difloxacin	10.2	4.4	9.2	6.6	6.4	6.4	1.1
Delafloxacin	8.2	7.4	8.7	3.1	4.6	8.9	5.1
TMP	6.7	5.6	7.9	2	0.5	-0.7	-1.7
CHL	12.3	9.5	11.8	5.5	7.3	3.2	3.2
DOX	6.8	8.5	7.9	7.2	9.1	7.3	4
ERM	-0.9	-0.4	-1.3	-6.8	-5	-1.3	-1.6
NOV	-1.9	1.5	4.3	-8.7	-4.6	3.6	0.9

A.

**MexF**	L683	T685	R690	F727	**S729**	E836	S81	T93	P136	D137	F295	V582	F584	Q586	**G626**	S633	Y671	A673	F675	P676	P677
**MexB**	L674	N676	D681	R716	**N718**	E825	S79	T91	K134	N135	K292	F573	Q575	Q577	**F617**	S624	M662	F664	F666	A667	P668

Access pocket (AP) residues

B.

**MexF**	R48	L90	T91	T130	**D132**	**P136**	L138	V141	Q178	L179	F180	G181	G183	N276	Q277	A279	L280	Y330	F619	G621	I624	G626	N629	F637
**MexB**	Q46	M88	T89	R128	**T130**	**K134**	F136	V139	Q176	V177	F178	G179	S180	Q273	D274	S276	I277	Y327	F610	V612	F615	F617	R620	F628

Distal pocket (DP) residues

Figure S1. Pairwise alignment of MexF and MexB reveals divergence at substrate recognition residues within the Access and Distal Pockets. Pairwise sequence alignment between MexF and its homolog MexB (*P. aeruginosa*) highlights non-conserved residues mapped to two critical substrate-interacting regions: the Access Pocket (AP) and Distal Pocket (DP). Residues in yellow represent positions in MexF that were selected for mutagenesis.

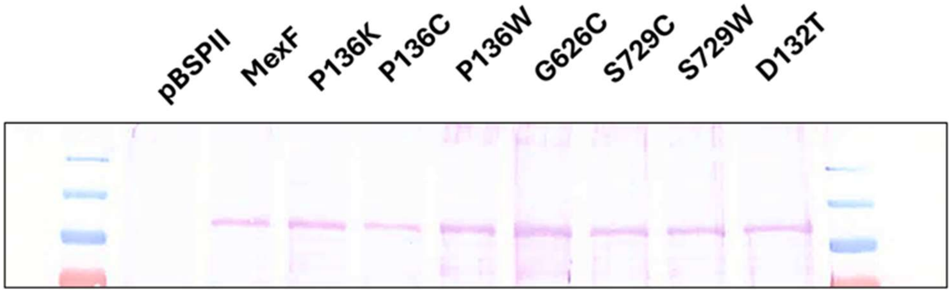

Figure S2. Expression analysis of MexF and its variants in P. aeruginosa Δ4-Pore cells. Whole-cell membrane fractions were prepared from P. aeruginosa Δ4-Pore cells harboring plasmids expressing wild-type MexF or the indicated point mutants (P136K, P136C, P136W, G626C, S729C, S729W, D132T). The pBSPII empty vector served as a negative control. Protein samples were resolved on SDS-PAGE and visualized by Western blot with anti-MexF primary antibody.

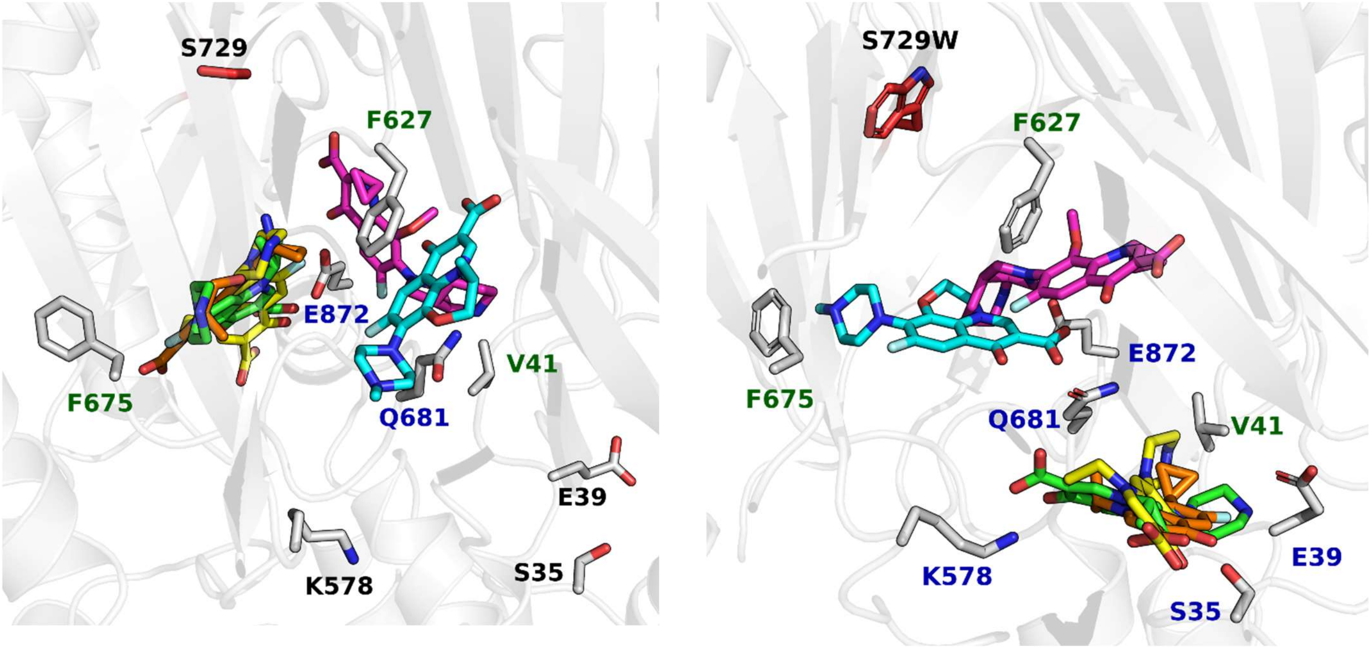

Figure S3. Representative docking poses of selected FQs within the access pocket of the wild-type (left) and mutated S729W (right) MexF structure (white cartoon and sticks, S/W729 in red). LEV is colored in cyan, MOX in magenta, CIP in green, NOR in yellow, and PAZ in orange. FQs that are not affected by the mutation (LEV and MOX) are located in the upper region of the pocket, both in the wild-type and in the mutated MexF structure. Conversely, those affected by the mutation (CIP, NOR, and PAZ) are located in the upper portion of the pocket in the wild-type, and in lower portion in the mutated form of the protein. This suggests that the substitution of a serine for a bulkier tryptophan pushes the affected antibiotics into the lower portion of the binding pocket, interacting with different residues (Ser35, Glu39, Val41, Lys578, and Gln681) than those lining the upper portion (Phe627, Phe675, and Glu872). Residue labels are colored according to the type of interaction (hydrophobic: green, polar: blue, no interaction: black).

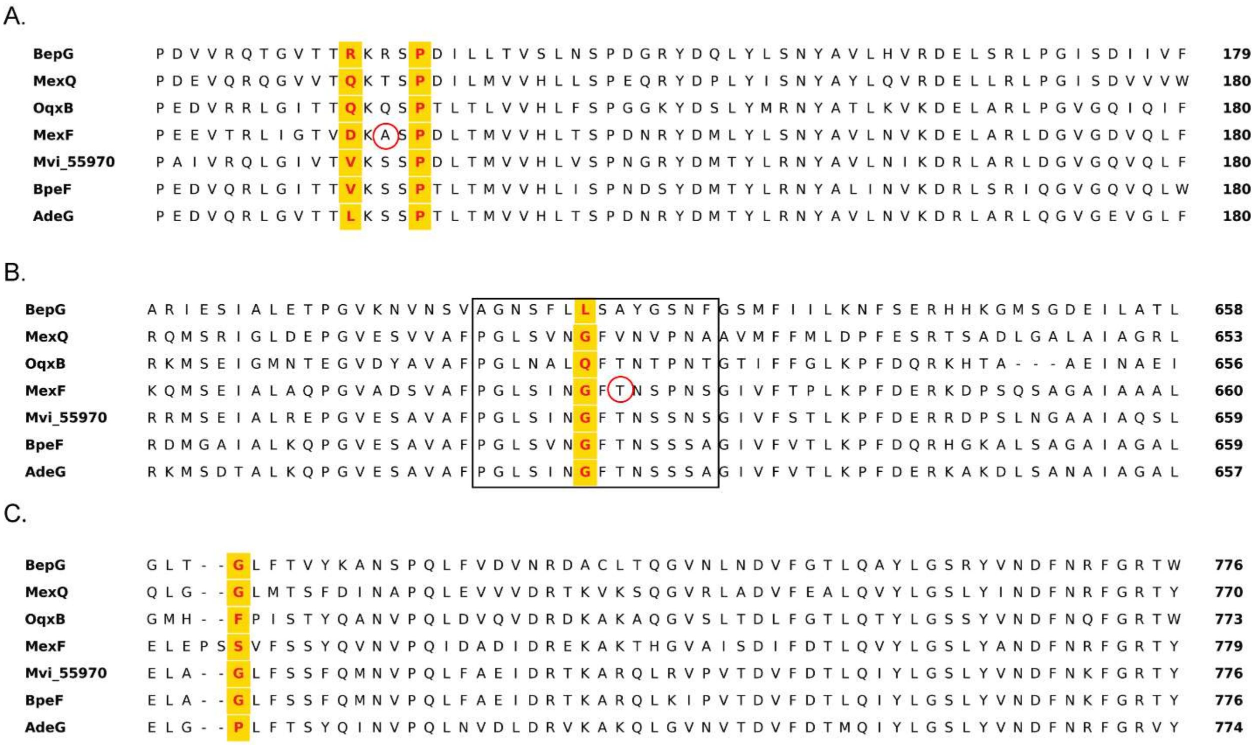

Figure S4. Multiple sequence alignment of the MexF mutated region with closest orthologs. Multiple sequence alignment was performed to compare the sequences with homologous RND transporters (sequence similarity >65%) from Gram-negative pathogens, including: M**exF** (*P. aeruginosa*, **BepG** (*Anatilimnocola aggregata*, A0A517YHT3), **MexQ** (*P. aeruginosa*, Q4LDT6), **OqxB** (*Salmonella enterica*, P95422), **Mvi_55970** (*Methylobacterium indicum*, A0A8H9C9V0), **BpeF** (*Burkholderia pseudomallei*, A0A0E1W0C1), and **AdeG** (*Acinetobacter baumannii*, A0A385EXQ9). The column highlighted in yellow with bold red are the corresponding MexF residues mutated in this study. The residues comprising G-loops are boxed. Red empty circles are mutations in *mexF* gene identified in *P. aeruginosa* clinical isolates.[1]

(1) Fernandes, S. E.; Ortega, H.; Vaillancourt, M.; Galdino, A. C. M.; Stotland, A.; Mun, K. S.; Aguilar, D.; Doi, Y.; Lee, J. S.; Burgener, E. B.; et al. Evolutionary loss of an antibiotic efflux pump increases Pseudomonas aeruginosa quorum sensing mediated virulence in vivo. *Nature Communications* **2025**, *16* (1), 8397. DOI: 10.1038/s41467-025-63284-7.
